# Stem Lettuce and Its Metabolites: Does the Variety Make Any Difference?

**DOI:** 10.3390/foods10010059

**Published:** 2020-12-29

**Authors:** Janusz Malarz, Klaudia Michalska, Anna Stojakowska

**Affiliations:** Maj Institute of Pharmacology, Polish Academy of Sciences, Department of Phytochemistry, Smętna Street 12, 31-343 Kraków, Poland; malarzj@if-pan.krakow.pl (J.M.); klaudiaz@if-pan.krakow.pl (K.M.)

**Keywords:** apocarotenoid, caffeic acid derivative, flavonoid, *Lactuca sativa*, lignan, megastigmane, sesquiterpene lactone, 1,2,3,4-tetrahydro-*β*-carboline-3-carboxylic acid

## Abstract

The objective of the present study was to characterize chemical composition of hitherto unexamined aerial parts of *Lactuca sativa* var. *angustana* cv. Grüner Stern. In contrast to leafy and head varieties of the lettuces, asparagus lettuce grown in Europe is much less studied. Fractionation of a methanolic extract from leaves of *L. sativa* cv. Grüner Stern, supported with HPLC/DAD and ^1^H NMR analysis, led to the isolation and/or identification of numerous terpenoid and phenolic compounds, including five apocarotenoids—(-)-loliolide, (+)-dehydrovomifoliol, blumenol A, (6*S*,9*S*)-vomifoliol, and corchoionoside C; three sesquiterpene lactones; two lignans—((+)-syringaresinol and its 4-*O*-*β*-glucoside); five caffeic acid derivatives; and three flavonoids. Some of the compounds, to the best of our knowledge, have never been isolated from *L. sativa* before. Moreover, monolignols, phenolic acids and a tryptophan-derived alkaloid were found in the analyzed plant material. Stems, leaves and shoot tips of the asparagus lettuce were examined to assess their phenolics and sesquiterpene lactone content as well as DPPH scavenging activity. Another stem lettuce—*L. sativa* var. *angustana* cv. Karola, two cultivars of leafy lettuces and one species of wild lettuce—*L. serriola*, were also examined as a reference material using HPLC/DAD. The results have been discussed regarding our previous studies and the literature data available.

## 1. Introduction

Lettuce (*Lactuca sativa* L.), one of the most popular leafy vegetables, is present in the market in a wide variety of cultivars, which differ from one another in their taste, color, texture, pathogen resistance, and value as a functional food. This diversity is connected with an array of specialized metabolites produced by the plants. Rapid development of hyphenated analytical techniques brought about an increase in number of metabolomic studies devoted to crop plants, including popular vegetables like lettuce [1,2,3,4,5,6,7,8]. The studies have been chiefly focused on polyphenols, especially flavonoids and hydroxycinnamates, which are believed to carry some health benefits [9]. Another group of widely investigated specialized metabolites produced by the plant are terpenoids, including carotenoids, pentacyclic triterpenes, and sesquiterpene lactones. The last ones are responsible for the bitter taste of lettuce as well as, to some extent, for inhibition of insect feeding [10,11,12,13]. Pharmacological studies proved that lactucin-type guaianolides isolated from lettuce demonstrated anti-inflammatory and antinociceptive activity [14,15]. Metabolomic studies on the cultivated lettuce plants revealed also the occurrence of lignans [2,3,4,6]. The group of specialized plant metabolites seems to be of importance as an estrogenic component of human diet. However, their content in some lettuce cultivars is probably too low to exert any significant effect on the consumer’s health [16].

Stem lettuce, also called asparagus lettuce (*Lactuca sativa* L. var. *angustana* Irish, synonym—var. *asparagina* Bailey) is popular in China as both vegetable and medicinal plant (Chinese lettuce, celtuce or “wosun”) [17]. The vegetable is currently much less known in European countries, although it has a history of cultivation in our region [18,19]. Until the middle of the 20th century, a local cultivar of the plant (*L. sativa* L. var. *angustana* cv. Cracoviensis; “głąbiki krakowskie”) was popular in Kraków and the surrounding area.

Specialized metabolites accumulated by aerial parts of the asparagus lettuce have not been examined in detail. To the best of our knowledge, only two studies concerning secondary metabolites from edible parts of celtuce have been published so far [17,20]. The paper by Han et al. [17] dealt with sesquiterpenoids from stalks of a Chinese cultivar of the vegetable (purchased on local market). Starkenmann et al. [20] investigated the compounds responsible for the specific smell of the cooked stalks of celtuce. The only paper on constituents of *L. sativa* var. *angustana* cv. Grüner Stern [21] revealed a great structural diversity of sesquiterpene lactones accumulated in roots of the plant. Some of the isolated lactones were new for the cultivated lettuces.

Not only the stalks but also the fresh leaves, which could be used as a component of salads, are the edible parts of the asparagus lettuce [19]. Thus, we decided to study chemical constituents of the leaves from *L. sativa* var. *angustana* cv. Grüner Stern and compare their phytochemical profile to those of the two contemporary cultivars of *L. sativa* and to that of its wild predecessor—*L. serriola* [22]. Moreover, we were interested in chemical differences in composition of extracts from different organs of the celtuce plant.

The present study was aimed at identification of hitherto not described specialized metabolites in the leaves of asparagus lettuce (cv. Grüner Stern) that are of putative value for both consumers and breeders. An attempt was also made to find chemical traits specific for this old cultivar.

## 2. Materials and Methods

### 2.1. Chemicals and Solvents

Chlorogenic acid (5-CQA, purity > 97% by HPLC), cichoric acid (DCTA, purity > 98%), luteolin-7-*O*-*β*-d-glucoside (purity ≥ 98%), and a standard sample of cynarin (1,3-DCQA, purity > 99% by HPLC) were purchased from Roth (Karlsruhe, Germany). Caftaric acid (CTA, purity > 97%), quercetin-3-*O*-glucuronide (miquelianin, purity ≥ 95%), Folin–Ciocalteu reagent, gallic acid (GA), 2.2-diphenyl-1-picrylhydrazyl (DPPH), and 6-hydroxy-2,5,7,8-tetramethylchroman-2-carboxylic acid (Trolox) were obtained from Sigma-Aldrich Co. (St. Louis, MO, USA). Luteolin-7-*O*-glucuronide (purity > 95%) was supplied by HWI pharma services GmbH (Ruelzheim, Germany). Samples of luteolin, quercetin-3-*O*-*β*-glucoside (isoquercitrin), quercetin-3-*O*-β-galactoside (hyperoside), 3,5-dicaffeoylquinic acid (3,5-DCQA), lactucin-type and zaluzanin C-type sesquiterpene lactones, (-)-loliolide, protocatechuic, and caffeic acids as well as monolignols were isolated in our laboratory from different plants of the Asteraceae family and identified by comparison of their spectral data with those found in the literature. CHCl_3_, EtOAc BuOH, and MeOH of analytical grade were purchased from Avantor Performance Materials S.A. (Gliwice, Poland). Water was purified by a Milli-Q system (Millipore Corp., Bedford, MA, USA). MeOH and MeCN of HPLC grade as well as formic acid and glacial acetic acid of analytical grade were purchased from Merck (Darmstadt, Germany).

### 2.2. General Experimental Procedures

Optical rotation was determined on a PolAAr31 polarimeter (Optical Activity Ltd., Ramsey, UK). NMR spectra were recorded either in CDCl_3_ or in CD_3_OD on a Bruker AVANCE III HD 400 (resonance frequency—400.17 MHz for ^1^H) (Bruker Corp., Billerica, MA, USA). Analytical RP-HPLC separations were performed either at 25 °C, on a Zorbax Eclipse XDB-C18 column 4.6 × 150 mm (Agilent Technologies, Santa Clara, CA, USA) or at 40 °C on a on a Kinetex XB-C18 column (4.6 × 250 mm, 5 μm; Phenomenex, CA, USA) using an Agilent 1200 Series HPLC system (Agilent Technologies) equipped with a Rheodyne manual sample injector, quaternary pump, degasser, column oven, and a diode array detector. Semipreparative RP-HPLC was performed on a Vertex Plus column (Eurospher II 100-5 C18, 8 × 250 mm) (Knauer GmbH, Berlin, Germany) eluted with H_2_O-MeOH mixtures at a flow rate of 1.0–2.0 mL min^−1^, using Knauer P4.1S pump coupled to a dual wavelength UV/VIS detector operating at 210 and 260 nm. Conventional column chromatography (CC) was carried out using Merck silica gel 60 (0.063–0.2 mm), Polyamide 6 (Sigma-Aldrich Co.), and Sephadex LH-20 (GE Healthcare, Uppsala, Sweden). Thin layer chromatography (TLC) was performed on Merck silica gel 60 (0.25 mm) precoated plates.

### 2.3. Plant Material

Aerial parts of *L. sativa* L. var. *angustana* cv. Grüner Stern were collected three times. First, in July 2014, the raw material for isolation work was harvested (leaves, stalks, and shoot tops from the flowering plants; voucher No 03/2014). Next, in June and July 2020, the plant material for HPLC/DAD analyses (leaves from 8 and 15 weeks old plants; voucher No 02/2020) was collected concomitantly with the leaves of three other *L. sativa* cultivars and the leaves of *L. serriola*. Seeds of *L. sativa* L. var. *angustana* cv. Grüner Stern were obtained from the Botanical Garden of the Bonn University (Germany). Seeds of *L. sativa* var. *angustana* cv. Karola, *L. sativa* cv. Great Lakes, and *L. sativa* var. *crispa* cv. Amerikanischer Brauner were purchased from the commercial growers. Seeds of *L. serriola* L., collected from the wild, were delivered by the Botanical Gardens in Münster (Westfäliche Wilhelms-Universität, Münster, Germany; voucher No 05/2020) and Nantes (Ville de Nantes, France; voucher No 04/2020). All plants were grown in the Garden of Medicinal Plants, Maj Institute of Pharmacology, Polish Academy of Sciences, Kraków, Poland, where the voucher specimens were deposited. Data on cultivation conditions (type of soil, average annual temperature, annual rainfall, and agrotechnical procedures applied) are described elsewhere [23]. The collected plant material was dried at room temperature under shade.

### 2.4. Isolation of Chemical Constituents from Leaves of L. sativa *L.* var. angustana cv. Grüner Stern

The dried plant material (378 g) was powdered and exhaustively extracted with 70% MeOH (4 × 1.5 L) at room temperature with shaking. The combined extracts were concentrated in vacuo providing c. 500 mL of an aqueous suspension. The suspension was successively extracted with *n*-hexane, CHCl_3_, EtOAc, and *n*-BuOH. The obtained organic extracts were evaporated under the reduced pressure to yield 3.62, 1.60, 1.76, and 8.56 g of the dry residue, respectively.

The CHCl_3_ extract (1.60 g) was subjected to CC on silica (28.0 g) using gradients of EtOAc in hexane (up to 100% EtOAc) and subsequently, MeOH in EtOAc (up to 50% MeOH) as elution systems. The separated fractions (50 mL each) were monitored by TLC and the relevant ones were combined. Elution with hexane-EtOAc (4:1, *v*/*v*) gave fractions 52–59 that were further separated by the preparative TLC on silica using hexane-EtOAc (3:2, *v*/*v*) as a mobile phase (two developments) to yield **1** (2.7 mg). Fractions 60–65, after preparative TLC (hexane-EtOAc, 3:2 *v*/*v*, two developments) were subjected to semipreparative RP-HPLC (H_2_O-MeOH, 2:3, *v*/*v*, 2 mL min^−1^) to give **2** (2.0 mg). From the fractions 93–97, eluted with hexane-EtOAc 7:3 (*v*/*v*) and initially purified by TLC (hexane:EtOAc, 1:1, *v*/*v*), after semipreparative RP-HPLC (H_2_O-MeOH, 2:3, *v*/*v*, 2 mL min^−1^), **3** (1.3 mg) and **4** (0.9 mg) were obtained. Fractions 103–111 (eluted with hexane-EtOAc 1:1 (*v*/*v*)) were further separated by preparative TLC (CHCl_3_-MeOH, 19:1, *v*/*v*) to furnish **5** (3.9 mg), and a mixture that was subjected to semipreparative RP-HPLC (H_2_O-MeOH, 3:7, *v*/*v*, 2 mL min^−1^) to yield **6** and **7** in a mixture (2:1, 1.3 mg). Elution with EtOAc-MeOH (9:1, *v*/*v*) gave fractions 175–184 that after preparative TLC (CHCl_3_-MeOH, 9:1, *v*/*v*) and subsequent semipreparative RP-HPLC (H_2_O-MeOH, 3:2, *v*/*v*, 2 mL min^−1^) yielded **8** (1.7 mg).

The EtOAc soluble part of the methanolic extract was partitioned by the conventional CC on silica gel. As an eluent, a gradient solvent system composed of MeOH in CHCl_3_ (up to 100% MeOH) was used. Fractions 12–29 (eluted with CHCl_3_-MeOH, 19:1, *v*/*v*) were subjected to preparative TLC to give subfractions A and B. The subfraction A was further separated by semipreparative RP-HPLC (H_2_O-MeOH-HCOOH-CH_3_COOH, 69:30:0.9:0.1, *v*/*v*/*v*/*v*, 2 mL min^−1^) to yield **9** (2.4 mg) and **10** (13.8 mg). The subfraction B after purification by semipreparative RP-HPLC (H_2_O-MeOH-HCOOH-CH_3_COOH, 49:50:0.9:0.1, *v*/*v*/*v*/*v*, 2 mL min^−1^) furnished **11** (2.6 mg). Fractions eluted with CHCl_3_-MeOH 9:1 (*v*/*v*) were submitted to preparative TLC (CHCl_3_-MeOH, 17:3, *v*/*v*) to give **12** (13.7 mg).

The *n*-BuOH part of the methanolic extract was initially separated by CC on polyamide to give fractions P1-P65 (100 mL each). The separated fractions were monitored by analytical RP-HPLC/DAD, and the relevant ones were combined. The fractions that contained commonly known *L. sativa* metabolites, easily detectable by HPLC/DAD (protocatechuic acid, caffeic acid, 5-CQA, luteolin-7-*O*-*β*-glucopyranoside, and isoquercitrin), as major constituents, were not further separated.

Fraction P1 (2.46 g), eluted with H_2_O, was subjected to CC on Sephadex LH-20 using H_2_O as an eluent. The obtained subfractions, P1S1–P1S2 (50 mL each) and P1S3-P1S12 (25 mL each), were monitored by RP-HPLC/DAD. The subfraction P1S4 was subjected to semipreparative RP-HPLC (H_2_O-MeOH-HCOOH-CH_3_COOH, 74:25:0.9:0.1, *v*/*v*/*v*/*v*, 1 mL min^−1^) to give a complex mixture of compounds containing (based on ^1^H NMR) benzyl glucoside, syringin, dihydrosyringin, roseoside, and cichorioside B (14.4 mg, *t*_R_ = 12.0 min) and a mixture of **13** and **14** (1:4, 8.8 mg, *t*_R_ = 24.2 min).

Fraction P2 (1.27 g), eluted with H_2_O, was separated on Sephadex LH-20, with H_2_O, to give subfractions P2S1–P2S12 (25 mL each). The subfraction P2S5 (23.4 mg), based on ^1^H NMR, contained esculetin glucoside (**15**) and syringaresinol glucoside as major constituents. The subfractions P2S7–P2S11 (13.8 mg) contained a tryptophan derivative (**16**).

Fractions P52–P55 (0.08 g), after semipreparative RP-HPLC (H_2_O-MeOH-HCOOH-CH_3_COOH, 59:40:0.9:0.1, *v*/*v*/*v*/*v*, 1 mL min^−1^), yielded luteolin-7-*O*-glucuronide butyl ester (4.5 mg). The compound, most likely, was an artifact formed during the separation process, as the corresponding peak was absent from the hydroalcoholic extract from leaves.

Fractions P57–P65 (0.16 g) were further purified by semipreparative RP-HPLC (H_2_O-MeOH-HCOOH-CH_3_COOH, 59:40:0.9:0.1, *v*/*v*/*v*/*v*, 2 mL min^−1^) to furnish a mixture of caffeoylquinic derivatives (**17** and **18**, 7.0 mg, 4:1, *t*_R_ = 15.2 min) and pure **18** (18.8 mg, *t*_R_ = 25.0 min).

### 2.5. Assessment of the Reducing Capacity of the Plant Material

The reducing capacity of the plant material, referred to as “total phenolic content” (TPC), was estimated using Folin–Ciocalteu colorimetric method, as described earlier [24]. Measurements were done using 20 mg of the dry plant material per sample. Leaves of 8- and 15-week-old plants of asparagus lettuce (cv. Karola and cv. Grüner Stern) and *L. serriola* (two accessions) were collected and dried separately for each individual plant. Results (means of three samples, each prepared from one plant) were expressed as gallic acid equivalents (mg GA g^−1^ DW).

### 2.6. DPPH Radical Scavenging Assay

Portions of dried and pulverized leaves, stalks, and shoot tops of *L. sativa* var. angustana cv. Grüner Stern (100 mg each) were extracted twice with 12.5 mL of 50% MeOH at room temperature. The solutions from two subsequent extractions were pooled together and evaporated in vacuo. The obtained residues were dissolved in 1 mL of 70% MeOH each, left to stand overnight, at 4 °C, centrifuged (11,340× *g*, 5 min), and the supernatant was diluted 10 times to obtain concentration corresponding to 10 mg of the dry plant material per 1 mL of the sample. DPPH was dissolved in methanol to obtain the stable free radical solution (100 µM). Solution (4 mM) of Trolox (reference compound) was prepared by dissolving of 100 mg of the compound in 100 mL of methanol. To a spectrophotometric cuvette (1 cm pathlength) containing 480 µL of the methanolic DPPH solution, 20 µL of the diluted plant extract (final concentration 10 mg DW mL^−1^) was added. A decrease in absorbance at λ = 517 nm was measured by UV/VIS CE 2021 spectrophotometer (Cecil, UK) after 0.5, 1, 2, 3, 4, 5, 10, 15, 20, and 30 min.

### 2.7. Sesquiterpene Lactone Analysis

Methanol extracts from the dry and pulverized plant tissues (200 mg) were subjected to RP-HPLC/DAD analysis, as it was described before [25]. Lactucin-like guaianolides could be easily detected in the extracts due to their distinctive chromophore (λ_max_—258 nm).

### 2.8. Quantification of Major Caffeic Acid Derivatives

The dry and pulverized plant tissue (50 mg) was extracted twice with 10 mL of 70% MeOH at room temperature for 3 h on a rotary shaker (100 r.p.m.). The extracts were combined and evaporated to dryness under reduced pressure to give a residue that was redissolved in 1 mL of 70% MeOH and centrifuged (11,340× *g*, 5 min) prior to HPLC analysis. Analytical RP-HPLC separations of the samples were performed as it was described earlier [26]. Quantification was carried out using an external standard method. The calibration curves were constructed using four concentration levels (0.001, 0.01, 0.1, and 1.0 mg mL^−1^) of 5-CQA, caffeic acid, CTA, and DCTA. Peak areas, measured at 325 nm, were referred to the corresponding calibration curve.

## 3. Results

**Figure 1:**
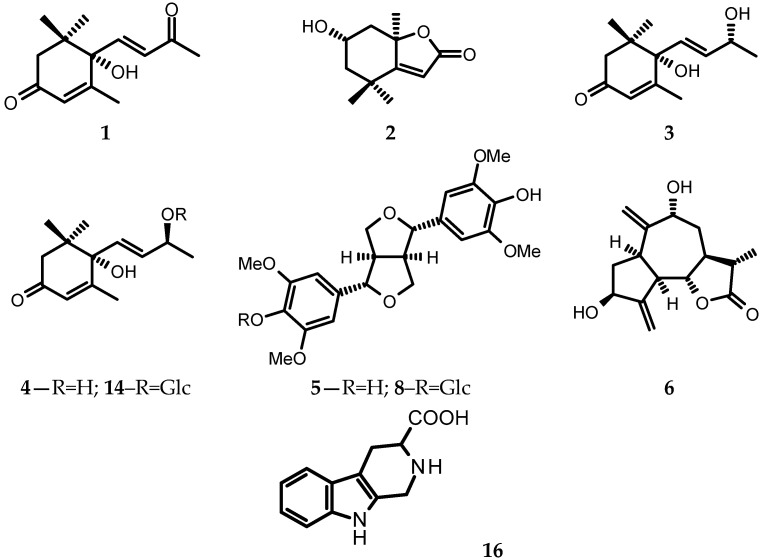
Chemical structures of some compounds isolated from leaves of the asparagus lettuce cv. Grüner Stern. Compounds **1**–**4** ((+)-Dehydrovomifoliol, (-)-loliolide, blumenol A, (6*S*,9*S*)-vomifoliol), **6** (9*α*-hydroxy-11*β*,13-dihydrozaluzanin C), and **14** (corchoionoside C) were known natural products newly found in the cultivated lettuce, whereas compounds **5** ((+)-syringaresinol), **8** ((±)-syringaresinol-4-*O*-*β*-glucopyranoside), and **16** (1,2,3,4-tetrahydro-*β*-carboline-3-carboxylic acid) were previously tentatively identified in various commercial cultivars of lettuce by means of ultra-high-performance liquid chromatography (UHPLC) with photodiode array (DAD) and mass detection (ESI/QTOF/MS – electrospray ionization/quadrupole time-of-flight mass spectrometry).

Eighteen known natural compounds (**1**–**18**) were isolated from the leaves of *L. sativa* var. *angustana* cv. Grüner Stern, collected at the beginning of flowering. Structures of the compounds (some shown in Figure 1) were confirmed by direct comparison of their spectral data (UV, ^1^H NMR) and optical activity with either that of the standard samples or that found in the literature (^1^H NMR spectra of the newly isolated compounds are available as the Supplementary Material attached to this paper). (+)-Dehydrovomifoliol (=(6*S*,7*E*)-6-hydroxy-4,7-megastigmadien-3,9-dione, **1**) [27], (-)-loliolide (**2**) [28], (6*S*,9*R*) vomifoliol (=blumenol A, **3**), (6*S*,9*S*)-vomifoliol (**4**) [29], and (6*S*,9*S*)-roseoside (=corchoionoside C, **14**) [30,31] (see Figure 1), as far as we are aware, have not been found in cultivated lettuce plants until now. Lignans: (+)-syringaresinol (**5**) and (±)-syringaresinol-4-*O*-*β*-glucopyranoside (**8**) [32,33] as well as a tryptophan-derived alkaloid—1,2,3,4-tetrahydro-*β*-carboline-3-carboxylic acid (=lycoperodine-1, **16**) [34,35,36] were tentatively identified in some cultivars of *L. sativa* using advanced analytical techniques [2,3,4,5,6]. A sesquiterpene lactone-9*α*-hydroxy-11*β*,13-dihydrozaluzanin C (**6**) [37] has been previously isolated from roots of *L. laciniata* Makino (synonym of *L. sativa* L.) [38] and roots of *Lactuca altaica* Fisch. & C.A. Mey (currently regarded as a synonym of *L. serriola* L.) [39], but has not been found neither in stalks of Chinese celtuce [17] nor in roots of *L. sativa* cv. Grüner Stern [21]. Thus, the compound **6** has been isolated from the commercial cultivar of lettuce for the first time. A dihydroderivative of **6**—9*α*-hydroxy-4*β*,11*β*,13,15-tetrahydrozaluzanin C (**7**) [40] has been described as a constituent of celtuce stalks [17], and it has been the only report on its occurrence in cultivated lettuce plants.

The remaining compounds: protocatechuic acid (=3,4-dihydroxybenzoic acid, **9**), caffeic acid (**10**), luteolin (**11**), isoquercitrin (=quercetin-3-*O*-*β*-glucopyranoside, **12**), benzyl-*O*-*β*-glucopyranoside (**13**), cichoriin (**15**), 3,5-DCQA (**17**), and 4,5-DCQA (**18**) [2,41,42,43] are commonly known metabolites of wild and cultivated lettuces. Moreover, the presence of syringin, dihydrosyringin, and cichorioside B, in the analyzed plant material, was confirmed based on the ^1^H NMR spectra of some partially purified fractions.

The reducing capacities of extracts from leaves of *L. sativa* var. asparagina cv. Grüner Stern, cv. Karola, and *L. serriola* plants of different provenience (all 8 weeks old) ranged from 36.363 ± 2.78 to 43.27 ± 1.79 mg g^−1^ GA eq for cv. Grüner Stern and *L. serriola* from France, respectively (Table 1). Statistically significant differences in TPC (one-way ANOVA, *p* < 0.05) between *L. serriola* and the two examined cultivars of stem lettuce were not found neither in 8 weeks old nor in 15 weeks old plants.

**Figure 2:**
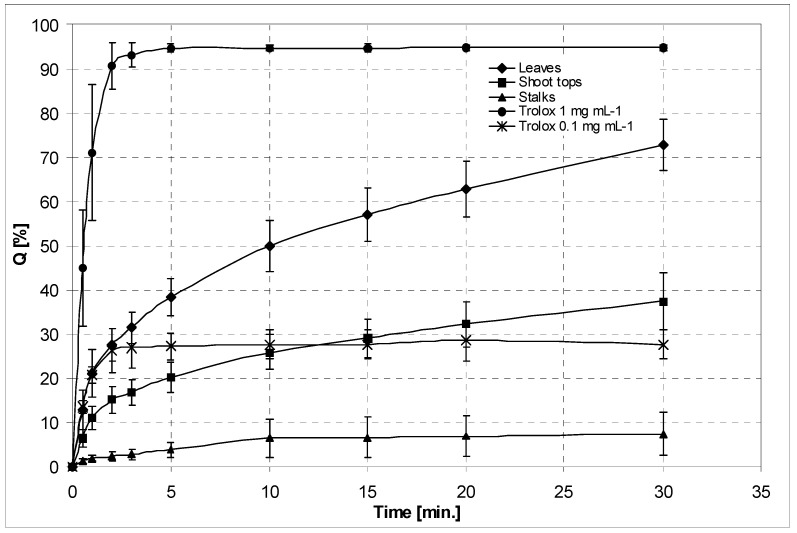
DPPH radical scavenging activity of 0.1 and 1 mg mL^−1^ solutions of Trolox (reference compound) and hydroalcoholic extracts from stalks, shoot tops, and leaves of *L. sativa* var. *asparagina* cv. Grüner Stern (10 mg of the dried plant material per 1 mL of extract).

In order to roughly assess radical-quenching activities of extracts from different parts of the asparagus lettuce, DPPH radical scavenging measurements were done. The experiments revealed substantial differences in activity of the examined extracts. The extract from leaves (10 mg DW mL^−1^) scavenged 72.7% ± 5.8% of the DPPH radical, whereas the extracts from stalks caused quenching of only 7.4% of the available free radical, after 30 min reaction (Figure 2).

Except for cichorioside B, identified as one of the components of the complex mixture of compounds from the butanolic fraction, we did not find any lactucin-like guaianolide during the fractionation of the extract from leaves of asparagus lettuce. Roots of the plant, investigated earlier [20], yielded mainly glucosides of **6** and 9*α*-hydroxyzaluzanin C accompanied by minor amounts of lactucin-like guaianolides, of which cichorioside B was the most abundant one. As 11*β*,13-dihydrolactucopicrin, another lactucin-like sesquiterpene lactone, was isolated from the celtuce stalks [17], its presence in the material under study was checked by HPLC/DAD method.

**Figure 3:**
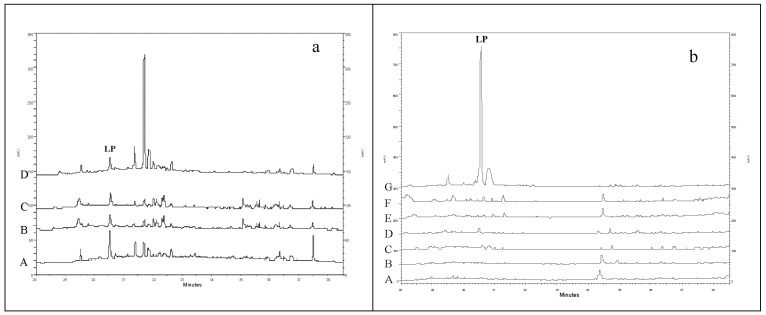
Chromatographic separations (HPLC/DAD, λ = 260 nm) of extracts from: (**a**) different parts/organs of *L. sativa* var. *angustana* cv. Grüner Stern, harvested in the beginning of flowering (A—roots, B—shoot tops, C—leaves, and D—stalks); (**b**) leaves of 8-week-old *L. sativa* and *L. serriola* plants (A—*L. sativa* cv. Grüner Stern, B—*L. sativa* cv. Karola, C—*L. sativa* cv. Great Lakes, D—*L. sativa* cv. Amerikanischer Brauner, E—*L. serriola* (Münster), F—*L. serriola* (Nantes), and G—leaves of *L. serriola* in flowering). Signals corresponding to 11*β*,13-dihydrolactucopicrin/lactucopicrin were marked as LP.

To assess the content of 11*β*,13-dihydrolactucopicrin/lactucopicrin (one of the most characteristic and easily detectable pairs of sesquiterpene lactones from *L. sativa*) in the analyzed plant material, a series of extracts was prepared from different organs of the asparagus lettuce (Figure 3a), leaves of *L. serriola*, and leaves of various cultivars of *L. sativa* (Figure 3b). The aerial parts of *L. sativa* cv. Grüner Stern, collected in July, contained c. 0.003% DW of 11*β*,13-dihydrolactucopicrin/lactucopicrin. The content was two times smaller than that found in roots of the plant. In general, *L. serriola* leaves, and leaves of the analyzed *L. sativa* cultivars, did not accumulate detectable amounts of the compounds until the eighth week of growth. The exceptions were two individual plants of *L. serriola* (Nantes) and one plant of the Amerikanischer Brauner cultivar (c. 0.005–0.009% DW). After 15 weeks of growth, 11*β*,13-dihydrolactucopicrin/lactucopicrin was not detected in leaves of asparagus lettuce (cv. Grüner Stern and cv. Karola), but was present in leaves of both *L. serriola* accessions (0.087–0.123% DW) and leafy cultivars of *L. sativa* (0.008–0.014% DW).

**Figure 4:**
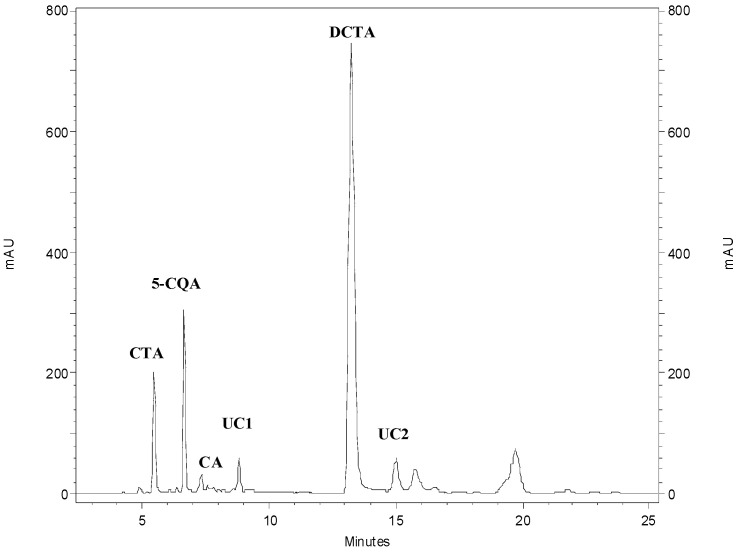
HPLC/DAD chromatogram of hydroalcoholic extract from leaves of 8-week-old *L. sativa* var. *asparagina* cv. Grüner Stern plant (50 mg of the dried plant material per 1 mL of extract, detection wavelength—325 nm). CTA—caftaric acid, 5-CQA—chlorogenic acid, CA—caffeic acid, UC1 and UC2—unidentified caffeic acid derivatives, DCTA—cichoric acid.

To investigate the diversity in polyphenolic profile among the analyzed plant species and cultivars, a series of hydroalcoholic extracts from leaf samples was prepared and chromatographically analyzed. Six major hydroxycinnamate signals (Figure 4) could be observed in the analyzed samples, i.e., CTA (*t*_R_ = 5.5 min), 5-CQA (*t*_R_ = 6.7 min), caffeic acid (*t*_R_ = 7.4 min), DCTA (*t*_R_ = 13.0 min), and two unidentified caffeates (*t*_R_ = 8.9 min and *t*_R_ = 15.1 min). The compounds were quantified according to the previously described procedure. The results are summarized in Table 2.

Looking at the obtained chromatograms, distinctly higher content of CTA in leaves of *L. serriola* in comparison with that in *L. sativa* cultivars could be noticed in the 8 weeks old plants. Statistical analysis (one-way ANOVA, *p* < 0.05 followed by Tukey’s HSD test) of the quantitative results obtained for caffeic acid derivatives (CTA, 5-CQA, caffeic acid, DCTA) confirmed significant differences in CTA content between *L. serriola* and cultivated lettuces (irrespectively of the cultivar). Seven weeks later, however, no differences in the major caffeate contents could be found (data not shown).

Major flavonoid signals were localized at *t*_R_ = 15.9 min (quercetin-3-*O*-*β*-glucoside, λ_max_—351 and luteolin-7-*O*-*β*-glucoside, λ_max_—345 nm, a pair of poorly separated compounds), *t*_R_ = 16.7 min (luteolin-7-*O*-glucuronide, λ_max_—345), and at *t*_R_ = 19.9 min (unidentified compound, λ_max_—351 nm). Virtually the same set of polyphenolics could be observed in every sample.

## 4. Discussion

Numerous factors regulate production and accumulation of secondary metabolites in lettuce including light, temperature, availability of nutrients, and presence of pathogens and pests. Accumulation of particular compounds in the plant organs could be also affected by the stage of a plant life cycle [20,21,22,23,24]. Moreover, colonizing microorganisms, e.g., mycorrhizal fungi, can affect secondary metabolism of the host plant [25]. The multiplicity of factors that affect the plant metabolome makes direct comparison of results obtained in different experiments difficult, though some general conclusions can be drawn on the basis of the available data.

The apocarotenoids **1**–**4** and **14** have not been found previously in cultivated lettuces. Comprehensive metabolomic fingerprints obtained by different HPLC/DAD/MS methods [2,5,6,7,8] did not reveal the presence of these compounds. Their absence from the investigated plant material, however, is very unlikely. The compounds originated from degradation of carotenoids and are widely distributed within the plant kingdom. Loliolide and *β*-damascenone are known metabolites of *L. serriola*, a wild predecessor of the cultivated lettuces [44,45]. Its plausible that the minute amounts of apocarotenoids were difficult to detect in a very complex matrix submitted to metabolomic analysis. Their isolation from the asparagus lettuce may suggest higher contents of the metabolites in the plant material under study in comparison with other lettuce cultivars. It is worth to note that lettuce is frequently used as a model plant to investigate allelopathic activity of apocarotenoids [46,47,48]. The compounds demonstrate diverse biological effects of ecological and pharmacological significance. (+)-Dehydrovomifoliol (**1**) inhibited germination of *L. sativa* cv. Roman and *Allium cepa* L. seeds and inhibited development of *L. sativa* cv. Napoli V.F. plants, but stimulated shoot and root elongation in *Hordeum vulgare* L. [46,47]. It moderately inhibited LPS-induced NO production in mouse RAW264.7 cells, after 24 h, by Griess reagent-based assay [49] and expressed cytotoxic effect against some cancer cell lines in vitro [50]. (-)-Loliolide (**2**), formerly identified as a potent ant repellent [51], recently has been postulated to be an endogenous inducer of herbivore resistance in plants [52]. The compound inhibited cellular senescence in human dermal fibroblasts [53], exerted antimelanogenic and oxidative stress-protective effects in mouse melanoma and human keratinocyte cells [54], inhibited HCV virus entry in vitro [55], and, similarly to **1**, LPS-induced NO generation in RAW264.7 cells [56]. Blumenol A (**3**) and its isomer (6*S*,9*S*)-vomifoliol (**4**) demonstrated anti-inflammatory activity in different types of in vitro assays [57,58,59]. Recently, neuroprotective function of vomifoliol in amyloid-*beta*_1-42_-treated neuroblastoma cells has been also studied [60]. Blumenol A moderately inhibited elongation of shoots and roots of *L. sativa* cv. Napoli V.F. and growth of some monocots [47,48]. Compound **5** (corchoionoside C, a glucoside of **4**) is one of the four stereoisomers of roseoside. Corchoionoside C inhibited histamine release, induced by antigen–antibody reaction, from rat peritoneal exudate cells [61]. Roseoside of unspecified stereostructure inhibited production of hypertension-related molecules by the rat myocardial cells stimulated with angiotensin II [62] and delayed carcinogenesis induced by peroxynitrite and TPA treatment in mice [63]. (+)-Roseoside caused vasorelaxation of the precontracted aortic rings from Sprague–Dawley rats (in endothelium-dependent manner) [64] and demonstrated insulinotropic activity [65].

Phenolic compounds are the most extensively studied lettuce metabolites. Their dietary intake is promoted as they are generally considered beneficial to human health [9]. According to van Treuren et al. [7], wild relatives of *L. sativa,* as well as primitive forms of domesticated lettuce, contain more polyphenols than modern cultivars. The main groups of the lettuce polyphenols are flavonoids (including anthocyanins from red varieties) and hydroxycinnamic acids derivatives and lignans. Although flavonoids and hydroxycinnamates are major constituents of lettuce plants (see DCTA content in leaves, Table 2), lignans are usually present in minute amounts. Up to 16 μg of lignans in 100 g of fresh lettuce leaves was reported by Milder et al. [16]. Isolation yield of lignans from the leaves of asparagus lettuce (c. 1.4 mg per 100 g dry weight) suggested relatively high content of the compounds in the investigated plant material. Metabolomic studies disclosed lignan accumulation patterns in some cultivated lettuces with syringaresinol and its glycosides as major representatives of this subclass of plant phenolics [2,3,4]. The asparagus lettuce investigated in the present study seems to follow the same scheme. In addition, major flavonoids detected in leaves of *L. sativa* cv. Grüner Stern corresponded to those found in leafy cultivars of the species. Significant qualitative and quantitative differences in the contents of the major hydroxycinnamates were not observed as well. The reducing capacity (TPC) of extracts from *L. serriola* and the two examined cultivars of the asparagus lettuce, Karola and Grüner Stern (Table 1), was comparable and falls within the range of TPC values estimated for different leaf cultivars of *L. sativa* [66]. The DPPH scavenging activity of extracts from different parts of *L. sativa* cv. Grüner Stern (Figure 2) suggests that the leaves of the plant are the richest in antioxidant compounds.

Sesquiterpene lactones are the most distinctive terpenoid constituents of lettuces. They are responsible for the bitter taste and, to some extent, for the insect resistance of the plants [10,11,12,13]. The compounds are usually present in very small amounts in the plant material intended for consumption (due to their bitterness) and their contents markedly increase at the bolting stage [67]. In the extract from leaves of the asparagus lettuce only 9*α*-hydroxy-11*β*,13-dihydrozaluzanin C (**6**) and 9*α*-hydroxy-4*β*,11*β*,13,15-tetrahydrozaluzanin C (**7**) were present in quantities that allow successful isolation of the lactones. The former compound was a novel constituent of the cultivated lettuce. Cichorioside B (dihydrolactucin glucoside) was identified as a component of a complex fraction from the butanolic part of the extract. Lactucopicrin and/or its dihydroderivative could not be detected in leaves of the asparagus lettuce collected in the initial phase of cultivation (until the 15th week). The compound was accumulated by the plants that enter the flowering stage albeit in small quantities.

Though metabolomic analyses of selected *L. sativa* cultivars [2,5,6] revealed the presence of tryptophan-derived alkaloids (including **16**), this is the first report on isolation of 1,2,3,4-tetrahydro-*β*-carboline-3-carboxylic acid (**16**) from the commercial cultivar of lettuce. The compound was first isolated from the leaves of *Allium tuberosum* [34]. Later on, it was purified from aged garlic, tomato, and some Asteraceae plants including *Cichorium endivia* L. [36]. 1,2,3,4-Tetrahydro-*β*-carboline-3-carboxylic acid induced apoptosis in colorectal cancer cell line HCT-8, in a dose-dependent manner [36], and was identified as one of the main antioxidants in aged garlic extracts [68].

## 5. Conclusions

Our findings concerning secondary metabolites from leaves of the old cultivar of stem lettuce (cv. Grüner Stern) broaden the knowledge on the chemistry of the garden lettuce—one of the most popular leafy vegetables. Among the newly identified constituents of the plant, apocarotenoids may have been of importance, taking into consideration both their ecological role and their pharmacological activity. It is worth to note that the apocarotenoids (compounds **1**–**4** and **14**) and 9*α*-hydroxyzaluzanin C derivatives (9*α*-hydroxy-11*β*,13-dihydrozaluzanin C and 9*α*-hydroxy-4*β*,11*β*,13,15-tetrahydrozaluzanin C) have not been found before in any of the commercial cultivars of the lettuce. The investigated plant material, in terms of polyphenolic content and antioxidative activity, was similar to modern leafy cultivars of *L. sativa*.

## Figures and Tables

**Table 1:** Reducing capacities (total phenolic contents) of extracts from leaves of *Lactuca sativa* var. *asparagina* and *Lactuca serriola* plants harvested after 8 and 15 weeks of growth in the open field. Results, expressed as gallic acid equivalents (GA eq), are means of three measurements (±SD).

Plant Material	Total Phenolic Content (mg g^−1^ Dry Weight) GA eq
*L. sativa* cv. Grüner Stern, 8 weeks	36.36 ± 2.78
15 weeks	44.09 ± 3.83
*L. sativa* cv. Karola, 8 weeks	41.55 ± 1.55
15 weeks	44.46 ± 3.82
*L. serriola* Münster, 8 weeks	36.38 ± 5.58
15 weeks	46.03 ± 4.47
*L. serriola* Nantes, 8 weeks	43.27 ± 1.79
15 weeks	46.81 ± 4.21

**Table 2:** Results of the assessment of major hydroxycinnamate constituents in the leaves of 8-week-old *Lactuca sativa* and *Lactuca serriola* plants. CTA—caftaric acid, 5-CQA—chlorogenic acid, DCTA—cichoric acid. Results are means of three independent measurements ± SD (results denoted with the same letter were not statistically different, *p* < 0.05).

Plant Material	CTA	5-CQA	Caffeic Acid	DCTA
*L. sativa* cv. Grüner Stern	0.121 ± 0.087 ^a^	0.108 ± 0.023 ^a^	0.018 ± 0.03 ^a^	1.023 ± 0.124 ^a^
*L. sativa* cv. Karola	0.185 ± 0.037 ^a^	0.246 ± 0.036 ^b^	0.021 ± 0.007 ^a^	1.733 ± 0.101 ^a^
*L. sativa* cv. Great Lakes	0.142 ± 0.01 ^a^	0.176 ± 0.047 ^a^	0.038 ± 0.001 ^a^	1.234 ± 0.026 ^a^
*L. sativa* cv. Amerikanischer Brauner	0.161 ± 0.02 ^a^	0.622 ± 0.035 ^c^	0.047 ± 0.01 ^a^	2.069 ± 0.075 ^a^
*L. serriola* Münster	0.560 ± 0.027 ^b^	0.062 ± 0.004 ^a^	0.029 ± 0.007 ^a^	1.888 ± 0.483 ^a^
*L. serriola* Nantes	0.363 ± 0.055 ^b^	0.243 ± 0.007 ^a^	0.027 ± 0.002 ^a^	2.267 ± 0.537 ^a^

## Data Availability

Data is contained within the article or supplementary material.

## Supplementary Materials

The following are available online at https://www.mdpi.com/2304-8158/10/1/59/s1, Supplementary material (Figures S1–S8, ^1^H NMR spectra of compounds **1**–**3**, **5**–**8**, **14**, and **16**).

## References

[1] Sobolev A.P., Brosio E., Gianferri R., Segre A.L. (2005). Metabolite profile of lettuce leaves by high-field NMR spectra. Magn. Reson. Chem..

[2] Abu-Reidah L.M., Contreras M.M., Arráez-Román D., Segura-Carretero A., Fernández-Gutiérrez A. (2013). Reversed-phase ultra-high-performance liquid chromatography coupled to electrospray ionization-quadrupole-time-of-flight mass spectrometry as a powerful tool for metabolomic profiling of vegetables: *Lactuca sativa* as an example of its application. J. Chromatogr. A.

[3] Viacava G.E., Roura S.I., Berrueta L.A., Iriondo C., Gallo B., Alonso-Salces R.M. (2017). Characterization of phenolic compounds in green and red oak-leaf lettuce cultivars by UHPLC-DAD-ESI-QtoF/MS using MS^E^ scan mode. J. Mass. Spectrom..

[4] Viacava G.E., Roura S.I., López-Márquez D.M., Berrueta L.A., Gallo B., Alonso-Salces R.M. (2018). Polyphenolic profile of butterhead lettuce cultivar by ultrahigh performance liquid chromatography coupled online to UV-visible spectrophotometry and quadrupole time-of-flight mass spectrometry. Food Chem..

[5] Yang X., Wei S., Liu B., Guo D., Zheng B., Feng L., Liu Y., Thomás-Barberán F.A., Luo L., Huang D. (2018). A novel integrated non-targeted metabolomic analysis reveals significant metabolite variations between different lettuce (*Lactuca sativa* L.) varieties. Hortic. Res..

[6] Ismail H., Gillespie A.L., Calderwood D., Iqbal H., Gallagher C., Chevallier O.P., Elliott C.T., Pan X., Mirza B., Green B.D. (2019). The health promoting bioactivities of *Lactuca sativa* can be enhanced by genetic modulation of plant secondary metabolites. Metabolites.

[7] Van Treuren R., Van Eekelen H.D.L.M., Wehrens R., De Vos R.C.H. (2018). Metabolite variation in the lettuce gene pool: Towards healthier crop varieties and food. Metabolomics.

[8] Qin X.-X., Zhang M.-Y., Han Y.-Y., Hao J.-H., Liu C.-J., Fan S.-X. (2018). Beneficial phytochemicals with anti-tumor potential revealed through metabolic profiling of new red pigmented lettuces (*Lactuca sativa* L.). Int. J. Mol. Sci..

[9] Crozier A., Jaganath I.B., Clifford M.N. (2009). Dietary phenolics: Chemistry, bioavailability and effects on health. Nat. Prod. Rep..

[10] Van Beek T.A., Maas P., King B.M., Leclercq E., Voragen A.G.J., De Groot A. (1990). Bitter sesquiterpene lactones from chicory roots. J. Agric. Food Chem..

[11] Mai F., Glomb M.A. (2016). Structural and sensory characterization of novel sesquiterpene lactones from iceberg lettuce. J. Agric. Food Chem..

[12] Rees S.B., Harborne J.B. (1985). The role of sesquiterpene lactones and phenolics in the chemical defence of the chicory plant. Phytochemistry.

[13] Daniewski W.M., Gumułka M., Drożdż B., Grabarczyk H., Błoszyk E. (1989). Sesquiterpene lactones. XXXVIII. Constituents of *Picris echioides* L. and their antifeedant activity. Acta Soc. Bot. Pol..

[14] Cavin C., Delannoy M., Malnoe A., Debefve E., Touche A., Courtois D., Schilter B. (2005). Inhibition of the expression and activity of cyclooxygenase-2 by chicory extract. Biochem. Biophys. Res. Commun..

[15] Wesołowska A., Nikiforuk A., Michalska K., Kisiel W., Chojnacka-Wójcik E. (2006). Analgesic and sedative activities of lactucin and some lactucin-like guaianolides in mice. J. Ethnopharm..

[16] Milder I.E.J., Arts I.C.W., van de Putte B., Venema D.P., Hollman P.C.H. (2005). Lignan contents of Dutch plant foods: A database including lariciresinol, pinoresinol, secoisolariciresinol and matairesinol. Br. J. Nutr..

[17] Han Y.F., Cao G.X., Gao X.J., Xia M. (2010). Isolation and characterization of the sesquiterpene lactones from *Lactuca sativa* L. var. anagustata. Food Chem..

[18] Lissek-Wolf G., Lehmann C., Huyskens-Keil S. (2009). Die Vielfalt alter Salatsorten—Eine Dokumentation.

[19] Kotlińska T., Rutkowska-Łoś A., Pająkowski J., Podyma W. (2015). Informator nt. Starych Odmian Roślin Rolniczych i Ogrodniczych Występujących na Terenie Rzeczpospolitej Polskiej i Możliwościach ich Introdukcji Do Uprawy Jako Odmiany Regionalne i Amatorskie. Ministerstwo Rolnictwa i Rozwoju Wsi.

[20] Starkenmann C., Niclass I., Vuichoud B., Schweizer S., He X.-F. (2019). Occurrence of 2-acetyl-1-pyrroline and its nonvolatile precursors in celtuce (*Lactuca sativa* L. var. *augustana*). J. Agric. Food Chem..

[21] Michalska K., Michalski O., Stojakowska A. (2017). Sesquiterpenoids from roots of *Lactuca sativa* var. *angustana* cv. Grüner Stern. Phytochem. Lett..

[22] De Vries J.M. (1997). Origin and domestication of *Lactuca sativa* L.. Gen. Resour. Crop. Evol..

[23] Piszczek P., Kuszewska K., Błaszkowski J., Sochacka-Obruśnik A., Stojakowska A., Zubek S. (2019). Associations between root-inhabiting fungi and 40 species of medicinal plants with potential applications in the pharmaceutical and biotechnological industries. Appl. Soil Ecol..

[24] Velioglu Y.S., Mazza G., Gao L., Oomah B.D. (1998). Antioxidant activity and total phenolics in selected fruits, vegetables, and grain products. J. Agric. Food Chem..

[25] Beharav A., Stojakowska A., Ben-David R., Malarz J., Michalska K., Kisiel W. (2015). Variation of sesquiterpene lactone contents in *Lactuca georgica* natural populations from Armenia. Gen. Resour. Crop. Evol..

[26] Malarz J., Stojakowska A., Kisiel W. (2013). Long-Term Cultured Hairy Roots of Chicory—A Rich Source of Hydroxycinnamates and 8-Deoxylactucin Glucoside. Appl. Biochem. Biotechnol..

[27] Kisiel W., Michalska K., Szneler E. (2004). Norisoprenoids from aerial parts of *Cichorium pumilum*. Biochem. Syst. Ecol..

[28] Sung P.J., Chen B.-Y., Chen Y.-H., Chiang M.Y., Lin M.-R. (2010). Loliolide: Occurrence of a carotenoid metabolite in the octocoral *Briareum excavatum* (Briareidae). Biochem. Syst. Ecol..

[29] Yamano Y., Ito M. (2005). Synthesis of Optically Active Vomifoliol and Roseoside Stereoisomers. Chem. Pharm. Bull..

[30] Çaliş I., Kuruüzüm-Uz A., Lorenzetto P.A., Rüedi P. (2002). (6S)-Hydroxy-3-oxo-α-ionol glucosides from *Capparis spinosa* fruits. Phytochemistry.

[31] Yajima A., Oono Y., Nakagawa R., Nukada T., Yabuta G. (2009). A simple synthesis of four stereoisomers of roseoside and their inhibitory activity on leukotriene release from mice bone marrow-derived cultured mast cells. Bioorg. Med. Chem..

[32] Xiong J., Bui V.-B., Liu X.-H., Hong Z.-L., Yang G.-X., Hu J.-F. (2014). Lignans from the stems of *Clematis armandii* (“Chuan-Mu-Tong”) and their anti-neuroinflammatory activities. J. Ethnopharmacol..

[33] Shahat A.A., Abdel-Azim N.S., Pieters L., Vlietinck A.J. (2004). Isolation and NMR spectra of syringaresinol-β-D-glucoside from *Cressa cretica*. Fitoterapia.

[34] Choi J.S., Kim J.Y., Woo W.S., Young H.S. (1988). Isolation of a *β*-carboline alkaloid from the leaves of *Allium tuberosum*. Arch. Pharm. Res..

[35] Ke R., Zhu E.-Y., Chou G.-X. (2010). A new phenylpropanoid glycoside from *Cirsium setosum*. Acta Pharm. Sin..

[36] Wang F.-X., Deng A.-J., Li M., Wei J.-F., Qin H.-L., Wang A.-P. (2013). (3S)-1,2,3,4-Tetrahydro-*β*-carboline-3-carboxylic acid from *Cichorium endivia* L. induces apoptosis of human colorectal cancer HCT-8 cells. Molecules.

[37] Kisiel W., Kohlmünzer S. (1987). Ixerin F from *Crepis biennis*. Planta Med..

[38] Nishimura K., Miyase T., Ueno A., Noro T., Kuroyanagi M., Fukushima S. (1986). Sesquiterpene lactones from *Lactuca laciniata*. Phytochemistry.

[39] Michalska K., Szneler E., Kisiel W. (2010). *Lactuca altaica* as a rich source of sesquiterpene lactones. Biochem. Syst. Ecol..

[40] Kisiel W., Barszcz B. (1996). Sesquiterpene lactones from *Crepis rhoeadifolia*. Phytochemistry.

[41] Kisiel W., Michalska K. (2002). A new coumarin glucoside ester from *Cichorium intybus*. Fitoterapia.

[42] Lee E.J., Kim J.S., Kim H.P., Lee J.-H., Kang S.S. (2010). Phenolic constituents from the flower buds of *Lonicera japonica* and their 5-lipoxygenase inhibitory activities. Food Chem..

[43] Luyen B.T.T., Tai B.H., Thao N.P., Cha J.Y., Lee H.Y., Lee Y.M., Kim Y.H. (2015). Anti-inflammatory components of *Chrysanthemum indicum* flowers. Bioorg. Med. Chem. Lett..

[44] Marco J.A., Sanz J.F., Albiach R. (1992). A sesquiterpene ester from *Lactuca serriola*. Phytochemistry.

[45] Abd-ElGawad A.M., Elshamy A.I., El Gendy A.E.-N., Al-Rowaily S.L., Assaeed A.M. (2019). Preponderance of oxygenated sesquiterpenes and diterpenes in the volatile oil constituents of *Lactuca serriola* L. revealed antioxidant and allelopathic activity. Chem. Biodiv..

[46] Macias F.A., Oliva R.M., Varela R.M., Torres A., Molinillo J.M.G. (1999). Allelochemicals from sunflower leaves cv. Peredovick. Phytochemistry.

[47] DellaGreca M., Di Marino C., Zarrelli A., D’Abrosca B. (2004). Isolation and phytotoxicity of apocarotenoids from *Chenopodium album*. J. Nat. Prod..

[48] Macias F.A., Lacret R., Varela R.M., Nogueiras C., Molinillo J.M.G. (2008). Bioactive apocarotenoids from *Tectona grandis*. Phytochemistry.

[49] Jin Q., Lee C., Lee J.W., Yeon E.T., Lee D., Han S.B., Hong J.T., Kim Y., Lee M.K., Hwang B.Y. (2014). 2-Phenoxychromones and prenylflavonoids from *Epimedium koreanum* and their inhibitory effects on LPS-induced nitric oxide and interleukin-1β production. J. Nat. Prod..

[50] Ren Y., Shen L., Zhang D.-W., Dai S.-J. (2009). Two new sesquiterpenoids from *Solanum lyratum* with cytotoxic activities. Chem. Pharm. Bull..

[51] Okunade A.L., Wiemer D.F. (1985). (-)-Loliolide, an ant-repellent compound from *Xanthoxyllum setulosum*. J. Nat. Prod..

[52] Murata M., Nakai Y., Kawazu K., Ishizaka M., Kajiwara H., Abe H., Takeuchi K., Ichinose Y., Mitsuhara I., Mochizuki A. (2019). Loliolide, a carotenoid metabolite, is a potential endogenous inducer of herbivore resistance. Plant Physiol..

[53] Yang H.H., Hwangbo K., Zheng M.S., Cho J.H., Son J.-K., Kim H.Y., Baek S.H., Choi H.C., Park S.Y., Kim J.-R. (2015). Inhibitory effects of (-)-loliolide on cellular senescence in human dermal fibroblasts. Arch. Pharm. Res..

[54] Park S.H., Choi E., Kim S., Kim D.S., Kim J.H., Chang S.G., Choi J.S., Park K.J., Roh K.-B., Lee J. (2018). Oxidative stress-protective and anti-melanogenic effects of loliolide and ethanol extract from fresh water green algae, *Prasiola japonica*. Int. J. Mol. Sci..

[55] Chung C.-Y., Liu C.-H., Burnouf T., Wang G.-H., Chang S.P., Jassey A., Tai C.-J., Tai C.-J., Huang C.-J., Richardson C.D. (2016). Activity based and fraction guided analysis of *Phyllanthus urinaria* identifies loliolide as a potent inhibitor of hepatitis C virus entry. Anivir. Res..

[56] Ren J., Qin J.J., Cheng X.R., Yan S.K., Jin H.Z., Zhang W.D. (2013). Five new sesquiterpene lactones from *Inula hupehensis*. Arch. Pharm. Res..

[57] Qin J.-J., Jin H.-Z., Zhu J.-X., Fu J.-J., Zeng Q., Cheng X.-R., Zhu Y., Shan L., Zhang S.-D., Pan Y.-X. (2010). New sesquiterpenes from *Inula japonica* Thunb. with their inhibitory activities against LPS-induced NO production in RAW264.7 macrophages. Tetrahedron.

[58] Dat N.T., Jin X., Hong Y.-S., Lee J.J. (2010). An isoaurone and other constituents from *Trichosanthes kirilowii* seeds inhibit hypoxia-inducible factor-1 and nuclear factor-κB. J. Nat. Prod..

[59] Zhou D., Wei H., Jiang Z., Li X., Jiao K., Jia X., Hou Y., Li N. (2017). Natural potential neuroinflammatory inhibitors from *Alhagi sparsifolia* Shap. Bioorg. Med. Chem. Lett..

[60] Tan M.A., Gonzalez S.J.B., Alejandro G.J.D., An S.S.A. (2020). Neuroprotective effect of vomifoliol, isolated from *Tarenna obtusifolia* Merr. (Rubiaceae), against amyloid-*beta*_1-42_-treated neuroblastoma SH-SY5Y cells. 3 Biotech..

[61] Yoshikawa M., Shimada H., Saka M., Yoshizumi S., Yamahara J., Matsuda H. (1997). Medicinal foodstuffs. V. Moroheiya. (1): Absolute stereostuctures of corchoionosides A, B, and C, histamine release inhibitors from the leaves of Vietnamese *Corchorus olitorius* L. (Tiliaceae). Chem. Pharm. Bull..

[62] Hong E.Y., Kim T.Y., Hong G.U., Kang H., Lee J.-Y., Park J.Y., Kim S.-C., Kim Y.H., Chung M.-H., Kwon Y.-I. (2019). Inhibitory effects of roseoside and icariside E4 isolated from a natural product mixture (No-ap) on the expression of angiotensin II receptor 1 and oxidative stress in angiotensin II-stimulated H9C2 cells. Molecules.

[63] Ito H., Kobayashi E., Li S.-H., Hatano T., Sugita D., Kubo N., Shimura S., Itoh Y., Tokuda H., Nishino H. (2002). Antitumor activity of compounds isolated from leaves of *Eriobotrya japonica*. J. Agric. Food Chem..

[64] Lee T.-H., Wang G.-J., Lee C.-K., Kuo Y.-H., Chou C.-H. (2002). Inhibitory effects of glycosides from the leaves of *Melaleuca quinquenervia* on vascular contraction of rats. Planta Med..

[65] Frankish N., de Sousa Menezes F., Mills C., Sheridan H. (2010). Enhancement of insulin release from the *β*-cell line INS-1 by an ethanolic extract of *Bauhinia variegata* and its major constituent roseoside. Planta Med..

[66] Liu X., Ardo S., Bunning M., Parry J., Zhou K., Stushnoff C., Stoniker F., Yu L., Kendall P. (2007). Total phenolic content and DPPH radical scavenging activity of lettuce (*Lactuca sativa* L.) grown in Colorado. LWT.

[67] Assefa A.D., Choi S., Lee J.E., Sung J.-S., Hur O.-S., Ro N.-Y., Lee H.-S., Jang S.-W., Rhee J.-H. (2019). Identification and quantification of selected metabolites in differently pigmented leaves of lettuce (*Lactuca sativa* L.) cultivars harvested at mature and bolting stages. BMC Chem..

[68] Wang X., Liu R., Yang Y., Zhang M. (2015). Isolation, purification and identification of antioxidants in an aqueous aged garlic extract. Food Chem..

