# Supplementary material for: Stem Lettuce and Its Metabolites: Does the Variety Make Any Difference?
Source: Foods. 2020 Dec 29;10(1):59. doi: 10.3390/foods10010059 (PMC7824169; doi:10.3390/foods10010059)
Supplement: Supplementary file 1 [file foods-10-00059-s001.pdf]

SUPPLEMENTARY MATERIAL

Stem lettuce and its metabolites: Does the variety make any difference?

Janusz Malarz [1], Klaudia Michalska [1], Anna Stojakowska [1*]

[1] Maj Institute of Pharmacology, Polish Academy of Sciences, Department of Phytochemistry, Smętna Street 12, 31-343 Kraków, Poland.

*corresponding author
E-mail address: stoja@if-pan.krakow.pl

Figures:

Fig. S1. ^{1}H NMR spectrum of (+)-dehydrovomifoliol (**1**)
Fig. S2. ^{1}H NMR spectrum of (-)-loliolide (**2**)
Fig. S3. ^{1}H NMR spectrum of blumenol A (**3**)
Fig. S4. ^{1}H NMR spectrum of corchoionoside C (**14**) in a mixture with benzyl glucoside
Fig. S5. ^{1}H NMR spectrum of (+)-syringaresinol (**5**)
Fig. S6. ^{1}H NMR spectrum of (±)-syringaresinol-4-*O*-*β*-glucopyranoside (**8**)
Fig. S7. ^{1}H NMR spectrum of 9*α*-hydroxy-11*β*,13-dihydrozaluzanin C (**6**) in a mixture with 9*α*-hydroxy-4*β*,11*β*,13,15-tetrahydrozaluzanin C (**7**)
Fig. S8. ^{1}H NMR spectrum of 1,2,3,4-tetrahydro-β-carboline-3-carboxylic acid (lycoperodine-1, **16**)

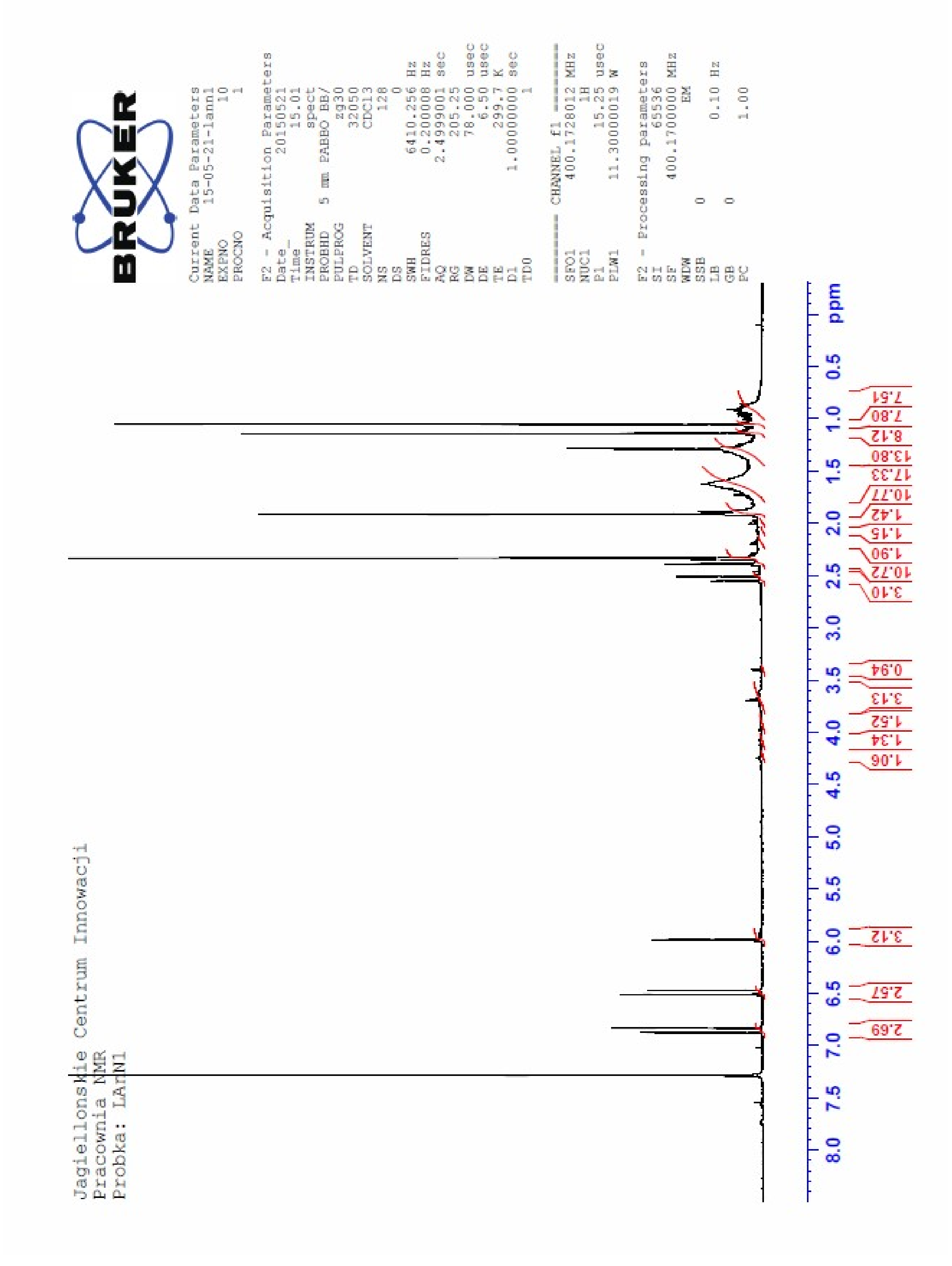

Fig. S1. ^{1}H NMR spectrum of (+)-dehydrovomifoliol (**1**)

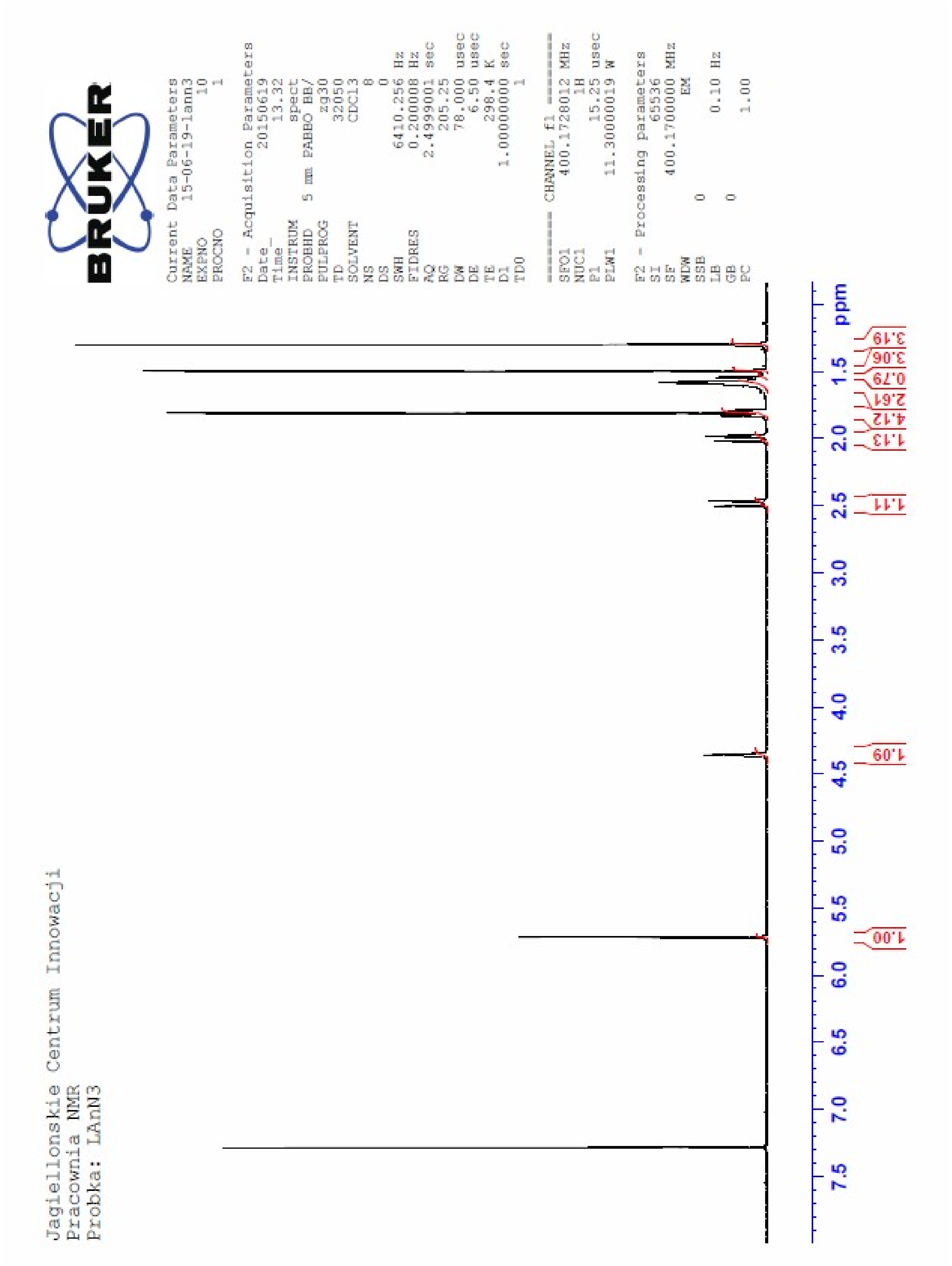

Fig. S2. ^{1}H NMR spectrum of (-)-loliolide (**2**)

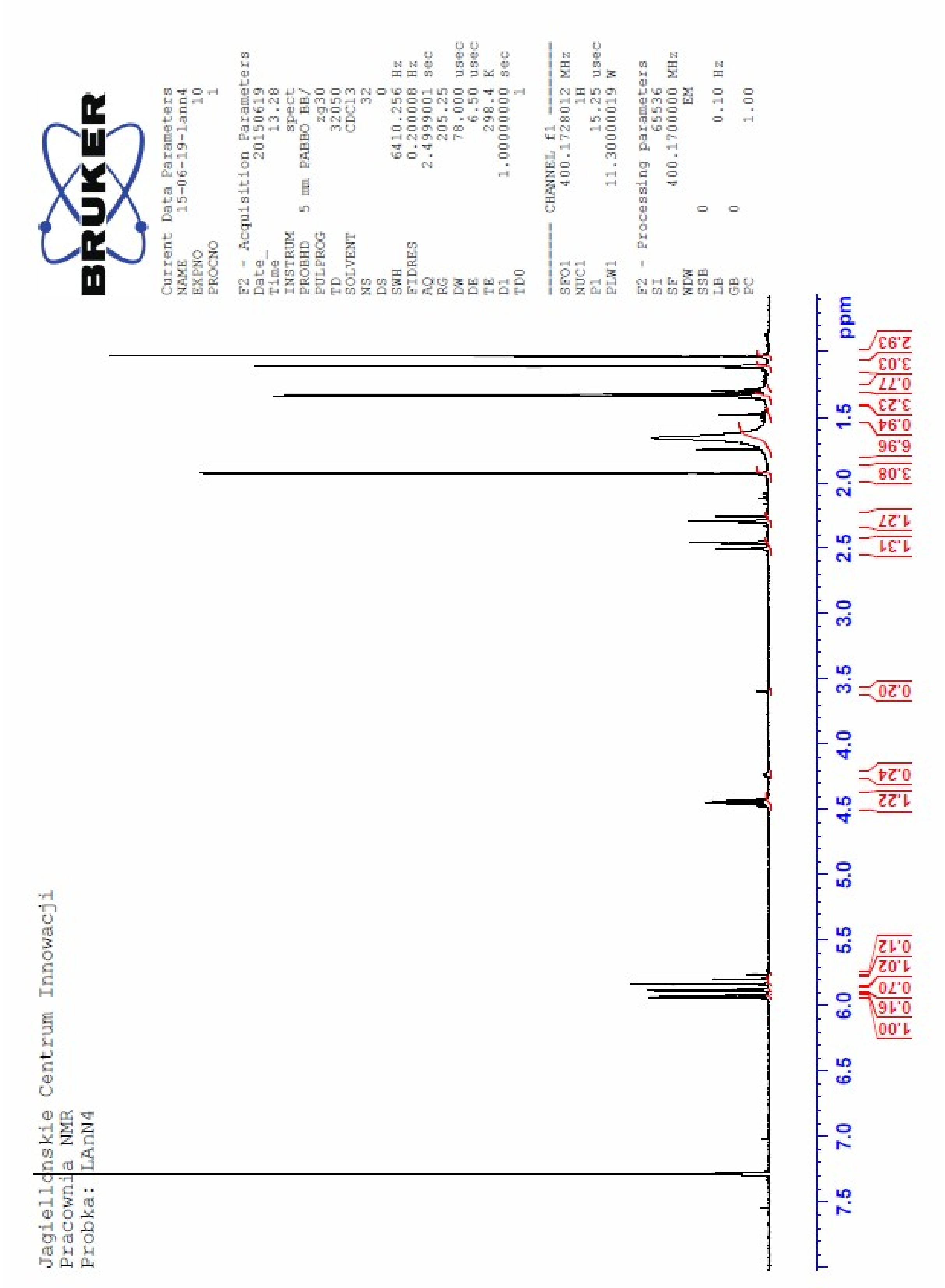

Fig. S3. ^{1}H NMR spectrum of blumenol A (**3**)

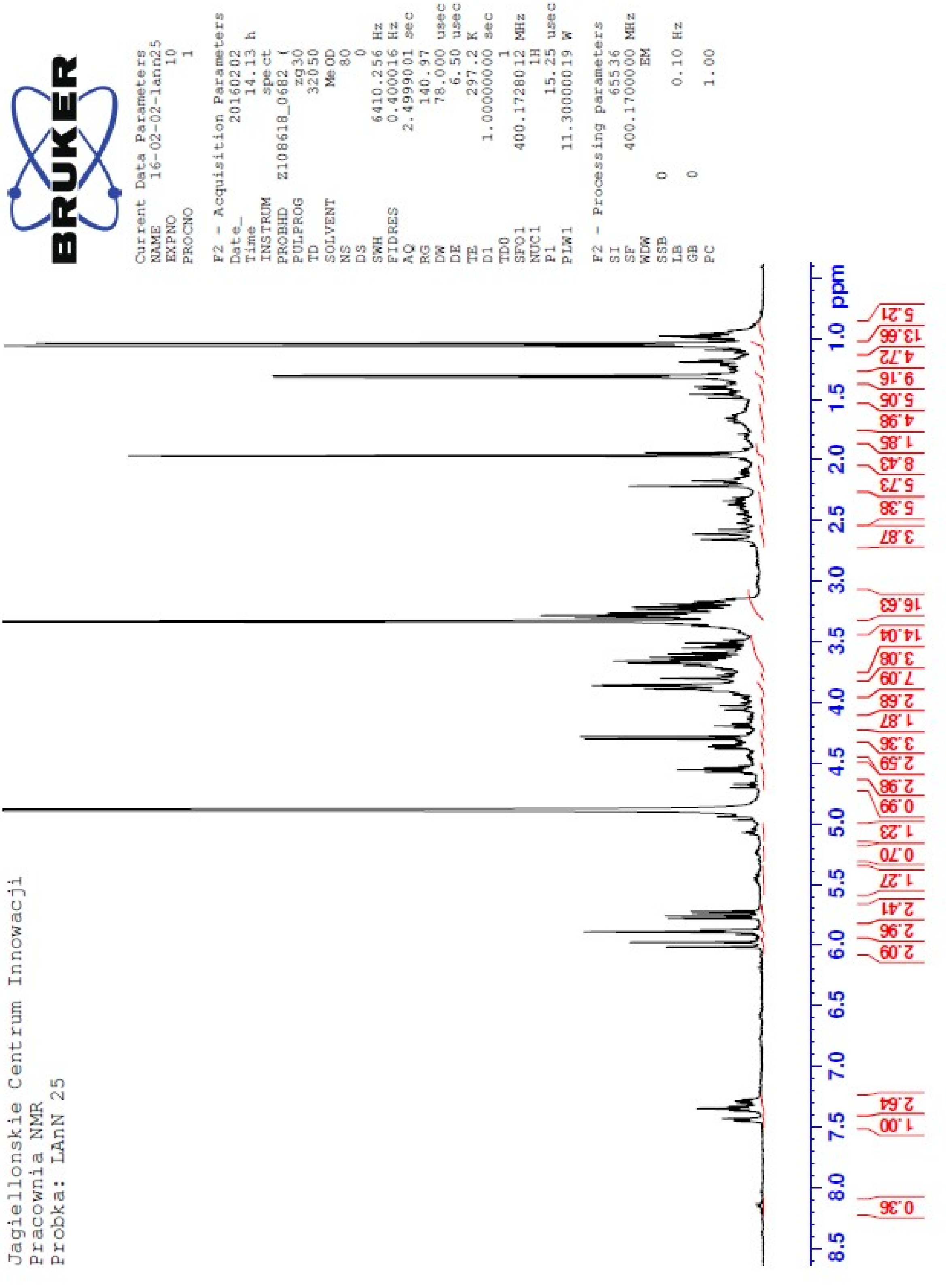

Fig. S4. ^{1}H NMR spectrum of corchoionoside C (**14**) in a mixture with benzyl glucoside

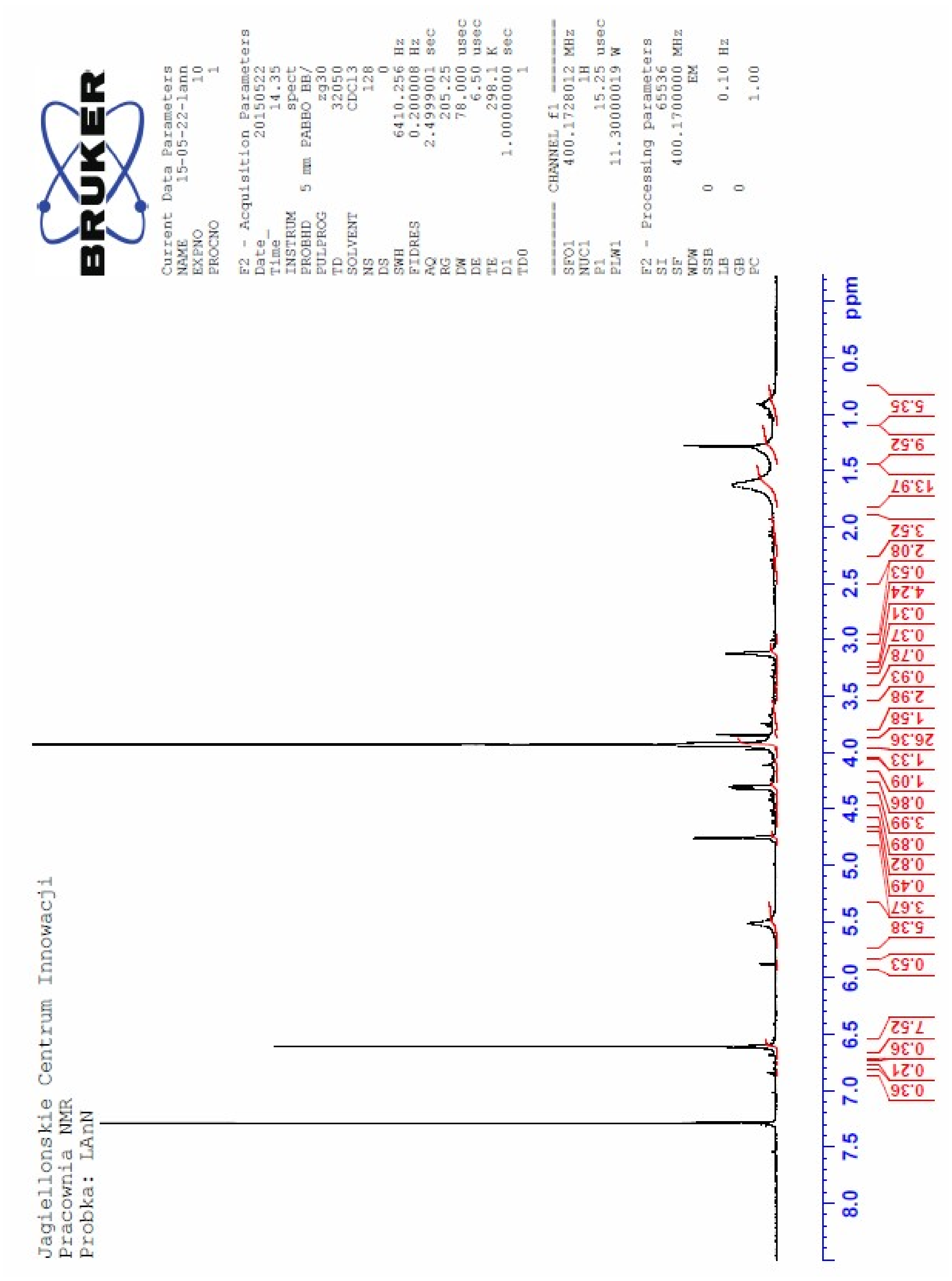

Fig. S5. ^{1}H NMR spectrum of (+)-syringaresinol (**5**)

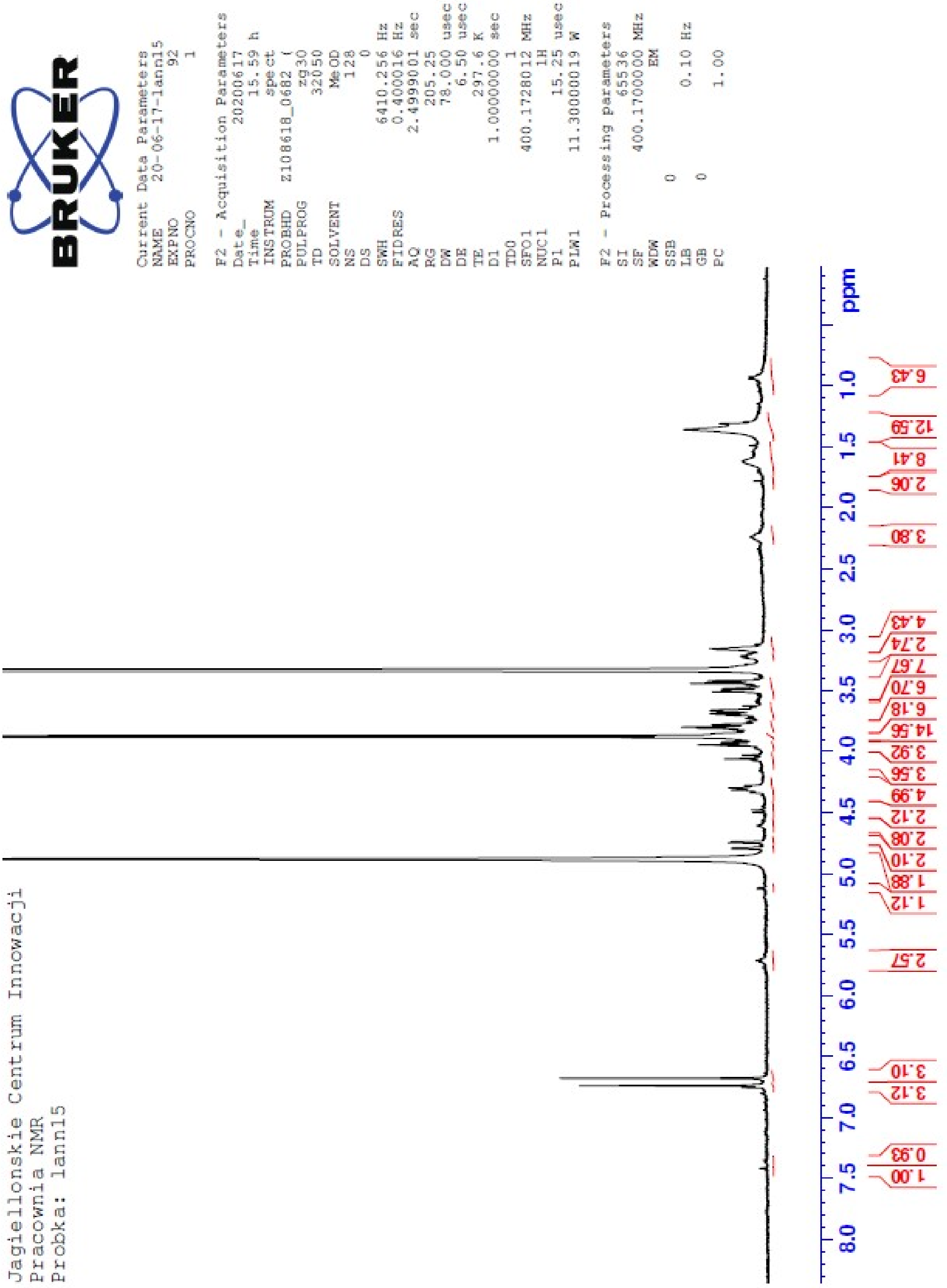

Fig. S6. ^{1}H NMR spectrum of (±)-syringaresinol-4-*O*-*β*-glucopyranoside (**8**)

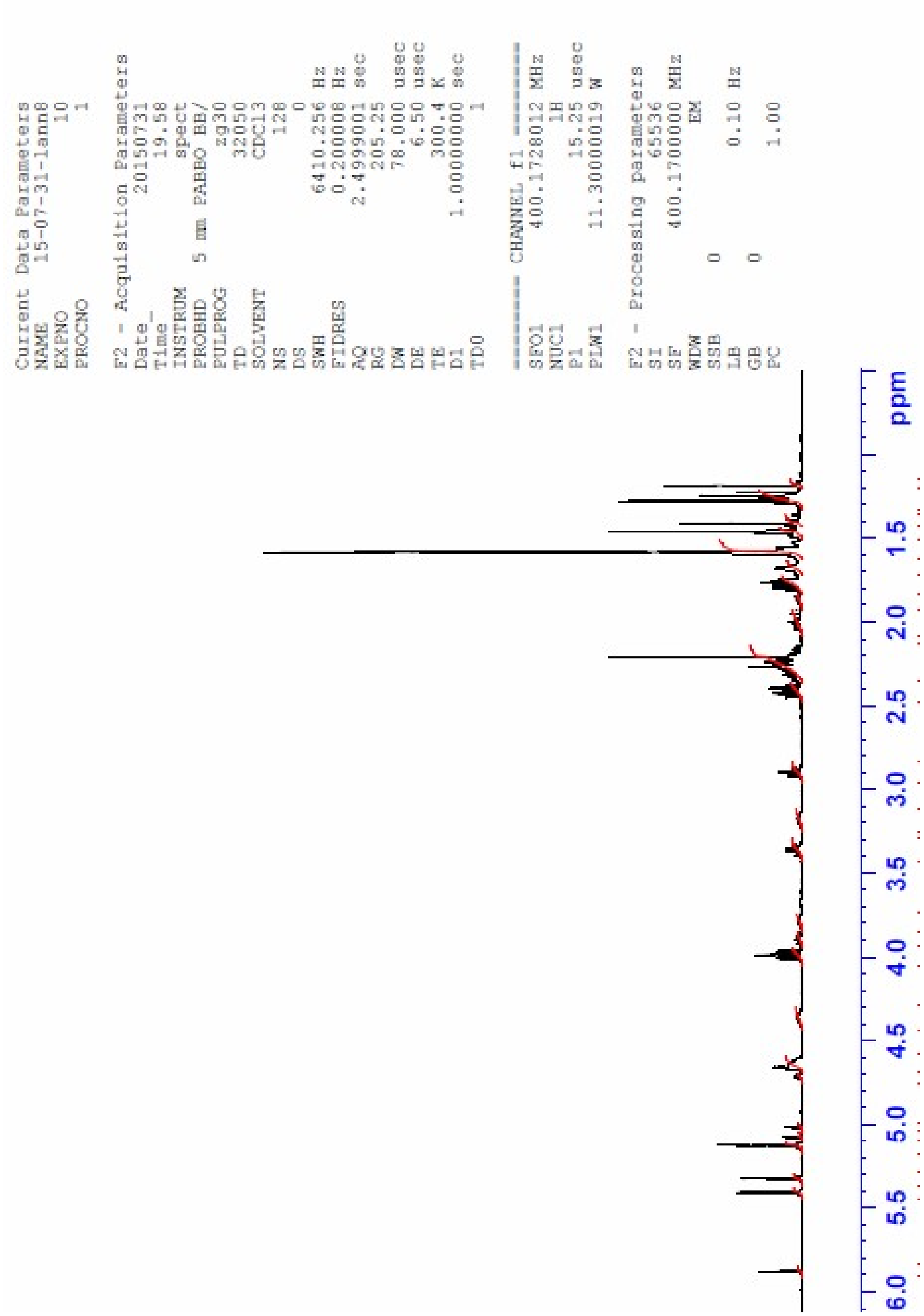

Fig. S7. ^{1}H NMR spectrum of 9α-hydroxy-11β,13-dihydrozaluzanin C (**6**) in a mixture with 9α-hydroxy-4β,11β,13,15-tetrahydrozaluzanin C (**7**)

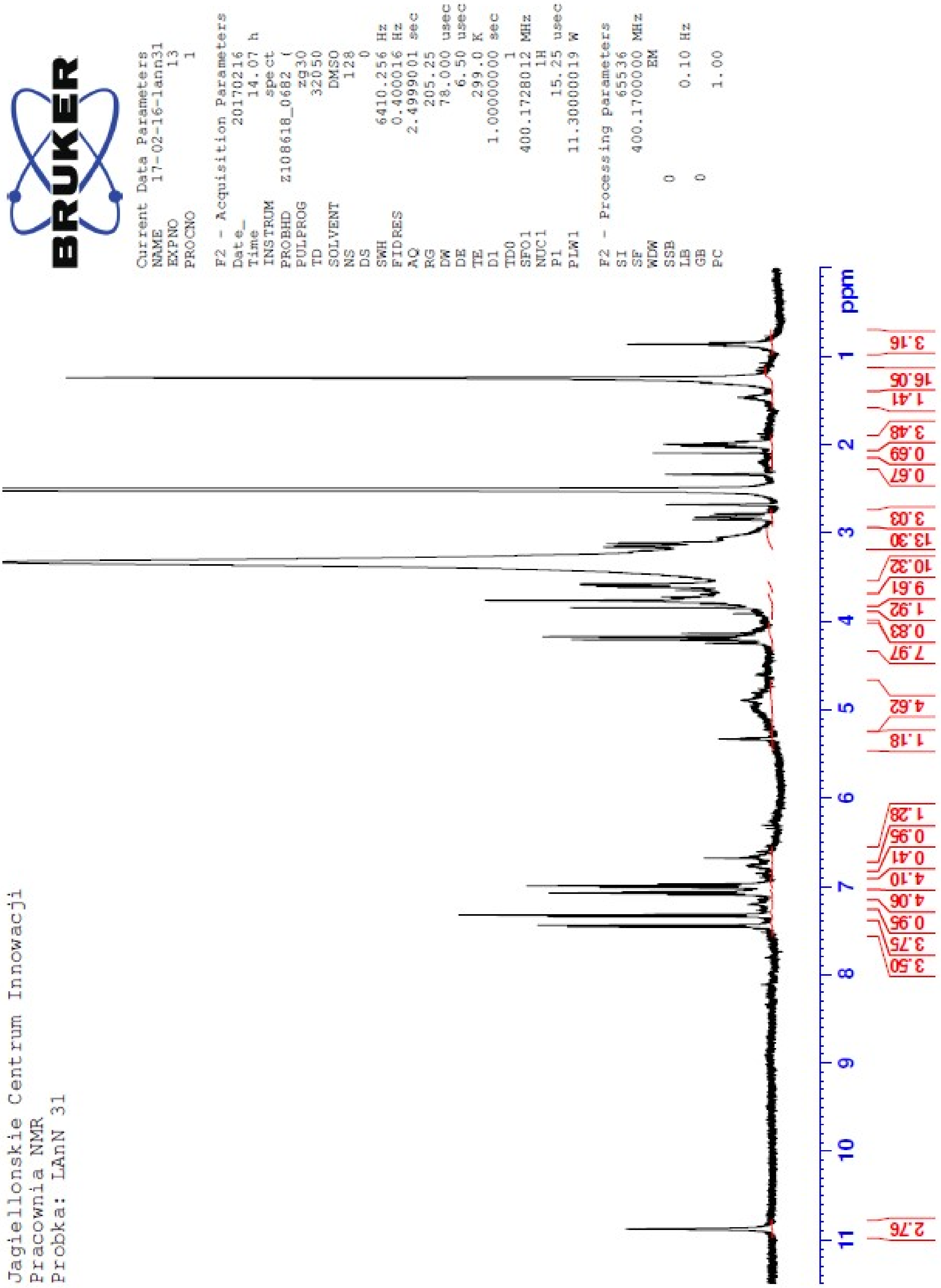

Fig. S8. ^{1}H NMR spectrum of 1,2,3,4-tetrahydro-β-carboline-3-carboxylic acid (lycoperodine-1, **16**)
